# Ross River Virus Infection: A Cross-Disciplinary Review with a Veterinary Perspective

**DOI:** 10.3390/pathogens10030357

**Published:** 2021-03-17

**Authors:** Ka Y. Yuen, Helle Bielefeldt-Ohmann

**Affiliations:** 1 School of Veterinary Science, The University of Queensland, Gatton, QLD 4343, Australia; k.yuen@uq.edu.au; 2 School of Chemistry and Molecular Science, The University of Queensland, St. Lucia, QLD 4072, Australia; 3 Australian Infectious Disease Research Centre, The University of Queensland, St. Lucia, QLD 4072, Australia

**Keywords:** epidemic polyarthritis, one health, mosquito-borne disease, zoonosis, climate change, arbovirus, equine, infectious disease, alphavirus

## Abstract

Ross River virus (RRV) has recently been suggested to be a potential emerging infectious disease worldwide. RRV infection remains the most common human arboviral disease in Australia, with a yearly estimated economic cost of $4.3 billion. Infection in humans and horses can cause chronic, long-term debilitating arthritogenic illnesses. However, current knowledge of immunopathogenesis remains to be elucidated and is mainly inferred from a murine model that only partially resembles clinical signs and pathology in human and horses. The epidemiology of RRV transmission is complex and multifactorial and is further complicated by climate change, making predictive models difficult to design. Establishing an equine model for RRV may allow better characterization of RRV disease pathogenesis and immunology in humans and horses, and could potentially be used for other infectious diseases. While there are no approved therapeutics or registered vaccines to treat or prevent RRV infection, clinical trials of various potential drugs and vaccines are currently underway. In the future, the RRV disease dynamic is likely to shift into temperate areas of Australia with longer active months of infection. Here, we (1) review the current knowledge of RRV infection, epidemiology, diagnostics, and therapeutics in both humans and horses; (2) identify and discuss major research gaps that warrant further research.

## 1. Introduction

Increasing frequency of extreme weather events over the last few decades across the globe has spiked concerns of the re-emergence of neglected pathogens, some with zoonotic potential. Climate change may have also potentially altered the disease dynamics of many vector-borne infectious agents as the vector population dynamics changes to adapt, such as Hendra virus transmitted by bats [1], and West Nile virus transmitted by mosquitoes [2]. The increase in rainfall and flooding events in recent years in Australia makes mosquito-borne diseases, such as Ross River Fever, likely to becoming more prevalent [3]. Ross River virus (RRV) infection is the most common human arboviral infection in Australia, with approximately 5000 cases notified annually [4,5].

Ross River virus belongs to the family *Togaviridae*, in the genus Alphavirus [6]. Other closely related viruses of veterinary and medical importance, include Chikungunya virus (CHIKV), Barmah Forest virus (BFV), Sindbis virus (SINV) and Getah virus (GETV) (Table 1). Alphaviruses, including RRV, are spherical, enveloped viruses with a single-stranded, positive-sense RNA genome of 10–12 kb [6]. The disease caused by RRV in humans is often referred to as “Ross River Fever” or “epidemic polyarthritis”. This review aims to provide a One Health perspective with a veterinary focus on the current knowledge of epidemiology, immunopathogenesis, diagnostics, and future forecast regarding RRV, and identify and discuss major knowledge and research gaps that warrant further research.

## 2. Epidemiology

Ross River virus is an arbovirus transmitted by mosquitoes, and is maintained in a marsupial–mosquito cycle. RRV was first isolated from mosquitos in the 1950s [7], then from a human [8] and a horse [9,10] in the early and late 1970s, respectively. RRV is endemic and enzootic in all states of Australia, Papua New Guinea and the Solomon Islands [4,5,11,12]. It was also isolated in New Caledonia [13], Fiji [14], American Samoa [15], and the Cook Islands [16] during the Pacific regional epidemic between 1979 and 1980.

### 2.1. Clinical Features in Humans

A wide range of non-specific symptoms can manifest in humans infected with RRV, varying from asymptomatic to prolonged, debilitating symptoms. In general, the incubation period for RRV ranges from 3 to 21 days, usually between 7 and 9 days [17,18]. For acute presentations, around 70–90% of patients experienced arthralgia (most commonly knee, ankle and wrist joints), lethargy or tiredness, and joint stiffness; 50–70% had myalgia; ~50% had skin rash, fever, and flu-like illness [19,20,21,22]. The severity and duration of clinical signs varies from a few weeks to a few months; however, more than 50% of patients continue to experience symptoms 12 months after initial presentation [19,21,22]. To date, there is no death reported that is directly attributed to RRV infection. However, it is worth noting that uncommon sequalae, such as encephalitis, have been reported [23]. Disease caused by other arthritogenic arboviruses, such as BFV and CHIKV can present with similar clinical signs in infected humans. The economic impact is estimated to cost Australia at least $4.3 million per year [24]. The estimated costs are likely to have been doubled in the financial year 2014–2015, when RRV cases nearly doubled as compared to previous years.

### 2.2. Clinical Features in Horses

Clinical signs in horses are not well documented as there are only a few reports published [25,26,27,28], making characterization of clinical features less well defined. The incubation period is not known. In general, clinical signs are non-specific, and range from lethargy to neurological (Table 2) [25,26,27,28]. RRV infection in horses is non-fatal; most horses recovered several months after initial presentation [25,26,27]. While an experimental study in the 1980s demonstrated only 1 out of 11 horses developing mild clinical signs [28], this finding should be interpreted cautiously. This may be due to the use of the prototype RRV T48 strain (T stands for Townsville, and 48 stands for mosquito pool number [18]) [7], originally isolated in the 1950s and since then undergone an unknown, but likely large number of in vitro passages in tissue cultures and in suckling mice over many years, which may have resulted in genomic and glycosylation changes that could influence virulence and pathogenesis.

### 2.3. Transmission Cycle

The RRV transmission cycle is complex, involving multiple hosts and vectors. It is believed the primary reservoir hosts are macropods, including wallabies and kangaroos [29,30,31]. It has been suggested that in areas where macropods are uncommonly found, for instance metropolitan or urban areas, brushtail possum (*Trichosurus vulpecula*) and humans are likely to be the main reservoir/spillover host [32,33]. Recent outbreaks in northeastern Australia also suggested a likely human–mosquito–human transmission cycle [34]. Horses and flying foxes are also considered as possible reservoir hosts in both peri-urban and rural areas as they are known to become viraemic after infection [28,32,35]. In contrast, dogs and cats remained aviraemic when experimentally infected with RRV and BFV, precluding transmission to mosquitoes and hence maintenance of transmission [36]. Unlike flaviviruses, the role of avian species in RRV transmission has traditionally been thought to be insignificant, but is somewhat debatable [37].

The role of horses in the transmission cycle remains unclear. Serological and molecular studies suggested that horses are unlikely to be a dead-end host and potentially could be an amplifying host for RRV during an epidemic [25,38]. The answer to this question is important to the equine industry, especially the racing industry, and people living in areas with a high density of horses, for example the Hunter Valley region in New South Wales, and outer suburbs of Greater Brisbane in Queensland. This will also affect the public health measures and intervention from the state governments to mitigate this most common arbovirus in Australia.

In order to maintain and complete the transmission cycle, the competent vector must be able to breed (requiring suitable environmental conditions, e.g., temperature), bite an infected reservoir host, ingest an infectious blood-meal, survive the extrinsic incubation period, all of which allow RRV to replicate and multiply to reach infectious titres, then bite another susceptible host. More than 40 mosquito species have been identified as competent vectors for RRV and this has been reviewed extensively elsewhere [5,18]. Briefly, the main mosquito vector varies depending on landscape, which can be divided into three areas; (1) Inland: *Culex annulirostris* (freshwater mosquito); (2) Metropolitan: *Aedes notoscriptus* (urban mosquito); (3) Coastal: *Ae. camptorhynchus* and *Ae. vigilax* (estuarine mosquitoes) [3].

### 2.4. Seroprevalence, Geographical Distribution and Risk Factors of RRV in Australia

In Australia, RRV infection distribution in humans varies spatiotemporally (Table 3). Queensland has consistently been the state with the highest number of reported cases, and the Northern Territory has had the highest notification rate (per 100,000) every year since 2010 (up to 2015) [4,39,40,41,42]. RRV transmission occurs throughout the year in the tropical coastal northeastern Australia with peaks associated with heavy monsoonal rainfalls [43,44,45]; in the subtropical Southeast Queensland area, RRV peaks between February and May [46]; the temperate area has epidemic activity, peaking between March and April, with sporadic cases reported at other times [44,47]. This is further evidenced by higher seroprevalence rates in blood donors in Northern Australia (22% in Townville; 15% in Darwin) as compared to Southern Australia (4% in Sydney; 0.84% in Melbourne) [48].

The epidemiology of RRV varies not only between states and territory but also into the local area level. Considering RRV is a mosquito-borne pathogen, this is not surprising as Australia is a large continent encompassing the tropical, subtropical and temperate areas, and with variations between coastal, grassland, rainforest and arid inland dessert within and between climate zones. Therefore, identifying generic risk factors to predict outbreaks at a local level has been difficult [49].

In spite of the difficulty in identifying generic risk factors, rainfall seems to be the most important environmental risk factor for RRV outbreaks across Australia [45,47,49,50,51,52]. Various studies found that there is a 0 to 3 month lag period between increased rainfall and RRV infections [45,47,50,51,53,54]. A study also found that notification rates in Queensland are twice as high in autumn compared to summer [55]. This could be explained by the time required for mosquitos to breed, then larvae to develop and hatch after heavy seasonal rainfall, which provides suitable breeding grounds such as floodwaters. The hatched mosquito then needs to enter the epizootic cycle of RRV transmission by ingesting a RRV-infectious blood meal from a reservoir host, which further adds to the lag period before infecting a human or other animal host with RRV. This sequence is evident in the finding that *Ae. camptorhynchus* larvae numbers peaked 2–3 months prior to the onset of RRV incidences [47]. On the other hand, frequent light rain might assist in adult mosquito survival and dispersal by replenishing existing breeding grounds and maintaining a high level of humidity [43]. The wide range of mosquito species involved in RRV transmission further complicates the underlying epidemiology. For example, abundance of freshwater mosquitos (e.g., *Culex annulirostris*) are mainly influenced by rainfall, whereas saltmarsh mosquitos (e.g., *Aedes vigilax*) are influenced by high tide [56].

Other climatic variables, such as tidal levels and temperatures, have also been identified as likely important environmental determinants for RRV transmission [45,47,50,51,53]. Landscape structure, such as river systems, floodwaters, wetlands, and marshlands, have been described in outbreak situations [49]. This is potentially further complicated by agricultural practices [46].

The notification case numbers in Table 3 do not reflect the true seroprevalence in the human population. Of all RRV infections, it was estimated that only 25–45% of people are symptomatic [14,18]; therefore, the true seroprevalence rate is much higher than the notification rate. Given the history of transfusion-transmitted arboviruses (such as West Nile virus [57,58]), this gives rise to the concern of RRV transmission via transfusion should a donor unknowingly donate blood during the viraemic phase of infection. The length of the viraemic phase of RRV infection in humans remains unknown; however, viraemia is detectable in mice 2–9 days post-infection [59]. This concern was justified when the first transfusion-transmitted RRV infection was reported in early 2014 after a patient received RRV-contaminated red blood cell component [60]. It is still not known whether the plasma and platelet component of the blood product from an infected donor is infectious, although it is highly likely to be the case [61,62]. While early modelling suggested that each year between 8 and 11 (or even higher) RRV-infected labile blood components are issued [48,63], the most recent study that accounted for the risk of RRV transmission in the areas where donor centers are located showed that the yearly risk of collecting an RRV-infected blood donation in Australia is much lower than previously reported (range from 1 in 2497 to 1 in 58,284 [48,59]) [64]. The results from this study are also more reliable in terms of detecting viraemia as it used RT-PCR to detect RRV viral RNA [64], a more sensitive and specific test than serological testing used in the earlier studies [48].

Serological surveys of RRV are not routinely performed in horses and it is not a notifiable disease of veterinary importance in Australia. Seroprevalence studies have reported as low as 26% to as high as 91% seropositivity in various regions of Australia (Table 4) [25,32,65,66,67]. During the 2011 epidemic of arboviral disease in Southeast Australia, high RRV seroprevalence was recorded in horses in Victoria and South Australia [68]. To the authors knowledge, Victoria is the only state in Australia that has an active surveillance program for equine arboviral diseases. Of the submitted samples for arbovirus investigation, incidence rate was approximately 30% between 2010 and 2013, and increased to 45% between 2013 and 2015 [26]. Government veterinary laboratories in Queensland and New South Wales focus their testing on flaviviruses, such as West Nile virus Kunjin strain and Murray Valley Encephalitis virus. The high ratio of seroconversion to disease reporting suggests most horses are likely to experience only subclinical or mild infection. This is of critical importance to the multi-million dollar horse racing industry as horses with mild RRV infection may go undetected. Therefore, the impact of even mild RRV infection on equine athletic performance must be considered. A relatively mild viral infection with little or no overt external evidence or clinical manifestations could result in limitations to performance that would cause a Thoroughbred racehorse to lose 1–2 s over 1200 m, which is equivalent to 8–10 m behind the expected position; an effect that could have serious economic implications for owners and trainers, and could adversely impact the future of the horse, with euthanasia a very real possibility. Similar considerations apply to other equestrian disciplines with highly demanding athletic performance, such as show-jumping in Standardbred horses.

## 3. Pathogenesis

After decades of research, the pathogenesis of RRV infection in humans remains unelucidated. Current knowledge of immunology and pathogenesis is mainly inferred from murine models. In veterinary medicine, apart from case reports/series, there is virtually no information regarding the disease pathogenesis and immunological responses in horses infected with RRV.

Peripheral blood leukocytes are among the first cells to encounter virus particles after a mosquito bite. However, the downstream innate immune response and cellular metabolism of peripheral blood mononuclear cells in humans and horses have not been established. Recently, a host cell molecule—Mxra8—has been found to facilitate cellular adhesion and entry in cell lines of mice, horse and human (fibroblasts, skeletal muscle cells, and chondrocytes) of multiple alphaviruses, including RRV [69,70]. The molecule is also required to establish infection in mice experimentally [69,70,71]. However, the physiological role of the Mxra8 receptor remains uncertain.

Earlier published studies focused on reporting the pathology of rash and synovium in human patients. Skin lesions, which rapidly recovered from rash, were dominated by CD8^+^ (T cytotoxic/suppressor) lymphocyte infiltration [72]; while synovial fluid from patients suffering from chronic arthritis had CD4^+^ (T helper/inducer) infiltration [73]. In both rash lesion and synovial fluid from human patients diagnosed with RRV disease, monocytes and vacuolated, phagocytic macrophages were also present, but the fluid contained no or low level of B lymphocytes and neutrophilic granulocytes [72,73,74,75,76]. Viral antigen and RNA were also demonstrated in synovial effusion, synovial tissues and skin lesions via immunolabelling and RT-PCR molecular testing [72,76,77]. The presence of viral components in these samples does not necessarily mean presence of actively replicating or intact virus, but could be due to residual viral components after ingestion by phagocytic cells. The latter is further supported by the lack of virion detection in electron microscopy [74,76]. Functional natural killer cells have also been detected in synovial fluid in a patient [75]. The lack of neutrophils and normal levels of complement component C3 and C4 in synovial effusion and serum suggested that immune complexes are not involved in the pathogenesis of RRV-induced arthritis [74,76]. Histological changes in the synovial lining tissue include hyperplasia, vascular proliferation, vascular congestion, fibrin deposition, and mononuclear cell infiltration (predominated by CD4^+^ T lymphocytes) [75,77]. In contrast to synovial fluid, mature B lymphocyte was detected in synovial tissues [77].

To date, there are no studies or case reports describing the muscle lesion from individuals experiencing myalgia. Studies found that RRV-induced myositis in experimentally infected mice had increased natural killer cells, monocytes, inflammatory macrophages (F4/80^+^), CD4^+^ and CD8^+^ T lymphocytes in skeletal muscles, but lacking CD19 reactivity (B lymphocytes) [78,79]. Histology showed extensive damage (myocyte necrosis, edema) to the striated muscle fibres with monocytes/macrophages infiltration with no evidence of infection of blood vessels [79,80]. However, these findings should be interpreted with caution as experimentally induced RRV infection in mice can produce severe morbidity, for instance, hindlimb paralysis, and can be fatal [79,80], which are not documented clinical outcomes in infected humans and horses, indicating possible differences in immunopathogenesis between the mouse model and natural hosts.

Generally speaking, in vitro studies in cell lines seem to support the above findings. A human synovial fibroblast cell line acutely infected with RRV in vitro demonstrated upregulation in monocyte chemoattractant protein-1 (MCP-1) mRNA expression [81,82], which, in turn, will cause monocytes to egress from the bone marrow and migrate into tissues, followed by maturation into macrophages at the site of infection. This is consistent with the predominant monocyte/macrophages cell population in synovium from acute synovitis. While interleukin-8 (IL-8) attracts and activates neutrophils, high levels of IL-8 can inhibit chemotaxis and activation of these cells. IL-8 mRNA expression was upregulated in human fibroblast by 8.2-fold as opposed to a 2.1-fold increase in a persistently infected murine macrophage cell lines [81]. While some suggested that the former might provide an explanation for the lack of neutrophils in acute synovial effusion, and the latter to the occasional low number of neutrophils in chronic synovial effusion, these observations must be interpreted with caution. It should be noted that these results are from two different cell types (fibroblast vs. macrophages), and two different host species (human vs. murine). Therefore, definitive interpretation and comparison of results should not be made.

Macrophages are derived from circulating monocytes after they leave the blood stream. The use of macrophage toxic agents, such as silica, prior to RRV experimental infection in mice resulted in diminished acute disease [79]. This supports the notion that macrophages participate in the pathogenesis of RRV disease. It has also been suggested that persistently infected macrophages may play a primary role in chronic or relapsing RRV disease. This is supported by: (1) in the murine macrophage cell line, level of RRV infection fell to a biologically undetectable level, then rose again in response to environmental changes and stress [82]; (2) detection of viral RNA in synovial samples in a small subset of infected humans five weeks after onset of symptoms [77]; (3) macrophages derived from human peripheral blood mononuclear cells after 24 h of cultures are able to be infected acutely with RRV [83]. However, it should be noted that the ability of macrophages to become infected persistently and productively with RRV has only been successfully achieved in a murine macrophage cell line (RAW 264.7) [79,82,83], and that viral antigen detection via immunolabelling has only been reported in skin and synovial samples collected during the early course of illness (within 7 days) [72,76]. Interestingly, monocytes from peripheral blood mononuclear cells of humans and a human monocyte cell line (Mono Mac 6) could not be infected with RRV, except in the presence of subneutralising levels of antibody [83].

Nevertheless, a few mechanisms have been suggested that could explain the ability of RRV to cause persistent infection in murine macrophages:

- Enhanced phagocytic activity of macrophages to remove damaged cells allows RRV to more readily establish persistent infection in macrophages [74,82].
- RRV dysregulates the mRNA expression of immunoregulatory co-stimulatory molecules (specifically down-regulation of CD80 and non-responsive CD86), and antiviral cytokines (specifically down-regulation of tissue necrosis factor-α and interferon-γ) [82], thus directly blocking the downstream antiviral adaptive T cell responses and allowing for persistence.
- Subneutralising level of antibody enables antibody-dependent enhancement of RRV infection via macrophage Fc receptors [83,84] by specifically disrupting transcription and translation of tissue necrosis factor and inducible nitric oxide synthase (i.e., key antiviral genes) through targeting the transcription factors IRF-1 and NF-κB, which then enables virus replication [84].

While monocytes/macrophages may play a part in the pathogenesis of RRV infection, these cells have also been demonstrated to have a protective role. Mice depleted of CCR2^+^ inflammatory monocytes suffered from more severe disease and high viral burden in tissues [85]. Type 1 interferon (IFN-1) is a major antiviral cytokine in mammals. It was demonstrated that IFN-1 produced and secreted by monocytes is upregulated upon RRV infection in a tissue specific manner [85,86]. The production of IFN-1 is mediated by retinoic acid-inducible gene 1-like receptors (RIG-1; a key sensor of virus infection) via the mitochondrial antiviral signaling pathway resulting in an increased mRNA expression of IRF3/IRF7 (a transcription factor that promotes IFN-1 production) [85,87]. Perhaps the persistence of infected monocyte/macrophages in the synovium caused prolonged production of IFN-1, which mediates the process of chronic/relapsing synovitis.

While the results generated from the murine model seems to explain the pathogenesis and underlying immunology of RRV infection in humans (and possibly horses), one should be mindful that specific features of the immune system differ between mice and humans [88]. Moreover, as mice are not natural hosts of RRV, it is probably also not the preferred animal model to study RRV pathogenesis and immunology in humans and horses. Nevertheless, results generated from previous studies may be used as a guide but should be interpreted cautiously in light of the differences between mice and humans, such that direct translation may not be possible.

## 4. Diagnostic Tests

Currently, diagnosis of infection from alphaviruses relies on traditional serological testing, namely enzyme-linked immunosorbent assay (ELISA) and virus neutralisation test (VNT). Rapid, high-throughput assays, such as polymerase chain reaction (PCR), have been developed but are not commercially available. Other assays, such as hemagglutination inhibition assay have also been used. Virtually none to very minimal information is available for veterinarians regarding the diagnostic tests available for RRV; therefore, this section focuses on the veterinary aspect with some mention of human diagnostics where appropriate.

### 4.1. Enzyme-Linked Immunosorbent Assay (ELISA)

Commercially available ELISA-based kits are used to detect RRV-specific IgG and IgM in human patients [89]. However, after a comprehensive literature search, no published articles were identified. A highly sensitive (97% in human; 100% in horses) and highly specific (98% in human; 95% in horses) epitope-blocking ELISA was developed in 2006 [90]. More importantly, no cross-reactivity was identified to closely related alphaviruses, such as BFV [90]. With the very promising results, one would expect the blocking ELISA assay to be put to use in research and clinical diagnostic work. However, to the authors’ knowledge, the blocking ELISA assay has not since been used for other similar types of research study nor is it commercially available.

ELISA was reportedly used to aid in diagnosing horses with RRV infection [91]. Paired blood samples are required, 2 to 4 weeks apart. Result interpretation can be complex (Table 5). Other limitations include the lack of sensitivity and specificity, and the potential cross-reactivity to other closely related viruses, for example, GETV and CHIKV, [92]. It is important to note that the results of this development have never been published in a peer-reviewed journal, and until recently, the ELISA test apparently had not been in use [91].

### 4.2. Virus Neutralisation Test (VNT)

Virus (or serum) neutralisation test (VNT or SNT) remains the gold standard for diagnosis and allows determination of antibodies titre levels. VERO cells (i.e., African green monkey (*Chlorocebus aethiops*) kidney fibroblast cell line) are frequently used. Theincubation period of the assay ranges from 4 to 6 days. The T48 prototype isolate is used for most VNT assays with a cut-off value for seropositivity between 1:10 and 1:40 [66,90]. Assay sensitivity and cut-off value differ between intra- and inter-laboratory depending on the cell type used (VERO vs. BSR cells), virus strain (T48 or other isolates), and other in-house variables. The detection of neutralising antibodies does not differentiate antibody subclasses, e.g., IgM and IgG, nor does it identify the source of antibody (i.e., maternal antibodies). In order to determine recent infection, a negative serum sample has to be available prior to the onset of clinical signs. Antibody cross-reactivity/protectivity against other closely related alphaviruses is possible, such as GETV [92], thereby reducing the specificity of this assay. While GETV does not circulate in Australia, other alphaviruses, such as BFV, do and could potentially give rise to false positives.

### 4.3. Haemagglutination Inhibition (HI)

Hemagglutination inhibition assay is no longer routinely performed and is infrequently used in either clinical diagnostic or research settings. A study evaluating the diagnostic performance of HI, blocking ELISA and VNT using animal sera found that HI is not a desirable testing method, and that VNT is slightly more sensitive than blocking ELISA [32].

### 4.4. Polymerase Chain Reaction (PCR)

Reverse transcriptase polymerase chain reaction (RT-PCR) has been developed as a rapid detection method for RRV infection in horses and appears to have high sensitivity and specificity [38]. However, the assay is not commercially available. A main limitation of the study was that only eight samples were tested. Furthermore, it is unknown for how long a horse may remain viraemic, nor is the time-relationship between viraemia and appearance of clinical signs known. Testing on a larger sample size would improve diagnostic and analytical sensitivity and specificity, which may help to better characterise the assay and potentially accelerate the process of commercialisation.

### 4.5. Virus Isolation

Virus isolation has low sensitivity and is often unsuccessful as viraemia is likely to have occurred prior to onset of clinical signs. This is further complicated by multiple factors, such as sample handling during transportation [93]. Therefore, virus isolation for RRV is infrequently performed for clinical diagnosis but rather for research purposes. Virus isolation was successful from horse blood [10,94] and serum [25].

## 5. Control, Prevention and Therapeutics

Prevention of RRV, and many other alphaviral infections are entirely based on general prevention of mosquito bites and management of mosquito breeding sites as there is currently no approved therapeutics to treat, nor registered vaccines to prevent, RRV infection in either humans or horses. Examples for humans include wearing light-colored, long-sleeved shirt, use of insect repellant and insecticide, and treatment of artificial containers, etc.

Treatments, in both human and veterinary medicine, are generally symptomatic, commonly in combination with non-steroidal anti-inflammatory drugs, and rest. Most recent research development in therapeutics is the use of PG545 (a heparan sulfate mimetic with anti-heparanase and anti-angiogenic effects [95]), which provided direct antiviral and anti-inflammatory effects when administered prophylactically and therapeutically in mice [96]. Another pharmaceutical candidate is pentosan polysulfate sodium, a very commonly used veterinary drug in dogs and horses suffering from osteoarthritis. Pentosan polysulfate sodium was reported to diminish the effect of RRV-induced joint pathology with chondroprotective effect and is currently in phase 2 clinical trial for use in human medicine [96,97].

A candidate formalin and UV light inactivated RRV vaccine for human use underwent clinical phase 3 trial in 2015 showed promising results [98]. However, there has not been an update issued since. In terms of veterinary use, two vaccinia-based vaccines, developed utilizing the “Sementis Copenhagen Vector” system and containing constructs of either RRV or a combination of CHIKV and Zika virus envelope protein, have recently been approved for clinical trial in horses against RRV infection [99,100,101].

Antibodies of alphaviruses may provide cross-protection against other closely related alphaviruses. Mice vaccinated with a commercially available formalin-inactivated, combined Japanese encephalitis plus GETV virus vaccine demonstrated partial cross-neutralisation against RRV, but interestingly not against CHIKV [102]. Similarly, RRV antisera from experimentally infected mice cross-neutralised GETV, and vice versa; but not CHIKV [92]. Therefore, future alphaviruses vaccine candidates should also be evaluated for the potential to cross-protect with other closely related alphaviruses. In a disease preparedness perspective, this will provide the chance to maximise the purpose of a vaccine, and reduce the time and costs associated with developing agent-specific vaccines.

## 6. Future Forecast

Most parts of the world are currently experiencing the effect of climate change as a result of human activities. Generally speaking, average ambient temperature and sea levels are rising, and the frequency of extreme weather events, for instance heavy destructive rainfall, and prolonged severe drought, are increasing as well. This would have implications for mosquito-borne diseases as the vector migrates and adapts to its environmental conditions, as for example seen for West Nile virus transmission across the world [103]. In addition, RRV has recently been suggested to have the potential to spread worldwide as an emerging infectious disease [104].

Climate warming means that the average temperature in Australia will continue to increase, coupled with increased frequency of extreme weather events, such as flooding, especially in years affected by the La Niña cycle [105,106]. This will likely shift the RRV infection dynamics slowly to the temperate region of Australia as the mosquito population dynamic shifts to adapt [107]. Therefore, temperate regions, such as Victoria, where RRV circulation status is currently a yearly epidemic may change to endemic as infection level increases. These areas may also see a lengthening of RRV active months, as temperature increases along with other climatic and risk variables prolong the suitable duration for mosquito activities. On the other hand, one would expect RRV infection may gradually decrease over time in the tropical areas, such as the Northern Territory. However, this depends on how well the mosquito vectors adapt to the upper thermal limits and whether new mosquito vector species are introduced or invade to allow RRV transmission to be sustained. Explosive outbreaks may occur in inland Australia that suddenly receive heavy rainfall after a prolonged drought period [24].

In order for the government to better implement public health measures and inform the public ahead of an outbreak of mosquito-borne disease, many studies in recent years have tried to develop predictive models. Use of traditional risk-factor-based epidemiological models, which assume that temperature and transmission are positively correlated, to predict future trend of mosquito-borne diseases may not be suitable and may potentially lead to misinformed public health intervention decisions being made. This is further evident by the fact that studies showed varying results [47,53,54,108,109]. Integration of statistical, mechanistic, and non-linear mixed models, for example characterization of non-linear thermal responses to identify transmission temperature optima and limits [107], would improve model prediction (recently reviewed in [110,111]).

A surveillance system is commonly used to monitor both exotic and endemic disease status and dynamics. Active surveillance by mosquito trapping in Western Australia seems to have proven useful to predict human RRV incidence [112]. This method, however, comes with a challenge: how many and where to set up trap sites? It is a difficult task to reach the optimal balance. Too many trap sites could lead to unnecessary costs and is labour-intensive, while too few trap sites would render intervention unsuccessful.

The use of sentinel animals is another appropriate surveillance method to monitor disease dynamics. In Northern Queensland and the Northern Territory, livestock, such as sheep and cattle are used as sentinel animals for the National Arbovirus Monitoring Program [113]. However, sheep, cattle, pigs and chicken are not suitable sentinels for RRV as they are not likely reservoir hosts for RRV transmission [52,65,94,114]. While horses are not used as sentinel animal in Australia, perhaps horses in the peri-urban interface would be a good model as sentinel for RRV and BFV at the very least. Classifying RRV infection as a notifiable disease of veterinary importance may also allow early detection of RRV outbreaks in humans.

The authors further suggest that horses arriving or returning from overseas should be tested for exotic alphaviruses infections during quarantine. The GETV outbreaks in Japanese racehorses [115,116] in recent years make incursion of exotic alphaviruses into Australia a very real possibility as high-performance Australian racehorses travel around the world to attend competitions, or overseas horses arriving Australia for competition or breeding purposes (artificial insemination is not allowed in the Thoroughbred industry in Australia). Recent laboratory transmission studies in mosquitoes for equine arboviruses continue to identify new mosquito species capable of transmitting exotic viruses [117]. This further supports the authors’ suggestion to strengthen mosquito-borne disease monitoring in horses to protect Australia from introducing zoonotic mosquito-borne diseases of both human and veterinary importance.

## 7. Gaps and Conclusions

The epidemiology and immunopathogenesis of RRV infection are complex and multi-factorial, involving multiple animal hosts and vector species, and are further complicated by climate change. The following major knowledge gaps have been identified:

- Pathogenesis and immunology of RRV infection in humans and horses remains unestablished. Results from the murine model may be used as a guide, but direct translation is not possible.
- Minimal diagnostics are available, especially in veterinary medicine. Current diagnostic tests would require weeks before the owner of the horse receives a definitive diagnosis of RRV. This timeframe would not be acceptable for racehorses as the value of the horse would decrease over the time of suspicion, with euthanasia being a very likely consequence.
- The lack of appropriate surveillance for endemic and exotic zoonotic equine arbovirus monitoring that is of human, livestock and veterinary importance.

To address the above issues, future studies utilizing an equine model would better elucidate the underlying immunopathogenesis of both humans and horses, and could potentially be used as a model for other infectious diseases; RT-PCR should be further validated to decrease turnaround time for reporting and improve animal welfare; horses should be considered as a sentinel animal model for zoonotic equine arboviruses to monitor disease dynamics and may potentially be used to inform public health measures. As internationalization continues and animal travel/movement becomes internationally more convenient, the potential for exotic zoonotic equine arboviruses incursion and the possibility of RRV emerges as an infectious disease worldwide should be considered seriously.

## Figures and Tables

**Table 1:** Classification of common alphaviruses.

Family	Genus	Disease Classification (Geographical Classification)	Examples	Commonly Used Abbreviations
*Togaviridae*	Alphavirus	Arthritogenic (Old World)	Barmah Forest virus	BFV
			Chikungunya virus	CHIKV
			Getah virus	GETV
			Ross River virus	RRV
			Sindbis virus	SINV
		Encephalitogenic (New World)	Eastern equine encephalitis virus	EEEV
			Venezuelan equine encephalitis virus	VEEV
			Western equine encephalitis virus	WEEV

**Table 2:** Summary of clinical findings of horses infected with Ross River virus naturally.

	Azuolas, J.K. et al. [25]	El-Hage, C.M. et al. [27]	Barton, A.J. et al. [26]
Study design	Prospective study	Case series	Longitudinal case series
Number of horses	750 ^1^	4	5
Location	New South Wales and Victoria	Bellarine Peninsula, Victoria	Lockyer Valley, Queensland
**Clinical signs**			
Lethary/exercise intolerance/poor performance	5/5	2/4	5/5
Pyrexia	—	3/4	2/5
Submandibular lymphadenopathy	—	4/4	—
Oral petechiae	—	2/4	—
Tachypnoea	—	—	3/5
Tachycardia	2/5	—	—
Muscle pain/stiffness	4/5	2/4	5/5
Lameness	4/5	1/4	2/5
Limb oedema	—	3/4	2/5
Synovial effusion	2/5	1/4	2/5
Inappetence/colic	1/5	¼	2/5
Ataxia/incoordination	4/5	—	—
**Haematology/Biochemistry**			
Hyperfibrinogenaemia	—	3/3	—
Hyperglobulinaemia	—	3/3	—
Neutropenia	—	—	1/5
Anaemia	—	—	1/5
Lymphocytosis	—	—	1/5
Lymphopenia	1/5		
Creatinine kinase	—	—	1/5
Aspartate aminotransferase	—	—	1/5
**Serology**			
IgM titre (range)	1:5120–1:81,920	1:5120–1:40,960	1:20,480
IgG titre (range)	0–1:40,960	0–1:10,240	1:20,480
Viral neutralization test (range)	—	—	1:160–1:2880

^1^ Only clinical sign from 5 horses; and serology from 3 horses were recorded.

**Table 3:** Spatio-temporal distribution of notified human cases of Ross River virus infection in Australia, by state or territory and financial year. The number of reported cases are in parenthesis.

State/Territory [Ref]	2010–11 [39]	2011–12 [40]	2012–13 [41]	2013–14 [42]	2014–15 [4]	2010–15
QLD	24.7% ^1^ (1397)	38.7% (1788)	43.7% (1683)	40.4% (1845)	63.2% (6371)	45.5% (13,084)
WA	14.6% (827)	33.2% (1533)	28.0% (1081)	32.5% (1485)	12.3% (1236)	21.4% (6162)
NSW	11.6% (658)	12.0% (556)	13.0% (502)	11.1% (509)	16.1% (1618)	13.4% (3843)
VIC	23.6% (1334)	5.9% (272)	4.9% (190)	3.5% (161)	3.4% (339)	8.0% (2296)
SA	20.4% (1154)	4.8% (222)	4.6% (177)	2.4% (111)	1.2% (119)	6.2% (1783)
NT	4.7% (263)	4.7% (219)	5.5% (211)	9.5% (434)	3.7% (374)	5.2% (1501)
TAS	0.2% (9)	0.4% (19)	0.2% (6)	0.4% (19)	0.0006% (6)	0.2% (59)
ACT	0.2% (11)	0.2% (8)	0.1% (5)	0.1% (5)	0.001% (11)	0.1% (40)
Total	100% (5653)	100% (4617)	100% (3855)	100% (4569)	100% (10,074)	100% (28,768)

QLD = Queensland; WA = Western Australia; NSW = New South Wales; VIC = Victoria; SA = South Australia; TAS = Tasmania; ACT = Australian Capital Territory; Ref = Reference. ^1^ % shown reflects distribution of nationally reported cases among each state or territory per year.

**Table 4:** Ross River virus serological surveys in horses in Australia.

Location	Year	Total Number of Horses	Sero-Positive Rate	Method	Reference
North QLD (total)	Sept 2013–June 2014	287	91%	VNT	[66]
Far North Coast to Tableland region (breakdown)		38	82%		[66]
Townsville to Burdekin region (breakdown)		201	95%		[66]
Mackay to Whitsunday region (breakdown)		49	86%		[66]
VIC, QLD, SA, NSW, WA	January–June 2011	982	21%	VNT or ELISA	[68]
VIC	October 2000–March 2002	750	56%	ELISA	[25]
Brisbane, QLD	November 1999	379	26%	VNT	[32]
South coast of NSW	1982–1983	120	62%	VNT	[65]
South coast of NSW	July–August 1980	23	65%	VNT	[67]

QLD = Queensland; WA = Western Australia; NSW = New South Wales; VIC = Victoria; SA = South Australia; TAS = Tasmania; ACT = Australian Capital Territory; VNT = Virus neutralization test; ELISA = Enzyme-linked immune-sorbent assay.

**Table 5:** Enzyme-linked immunosorbent assay (ELISA) interpretation for Ross River virus.

Scenario	Sample ^1^	IgM	IgG	Diagnosis	Comments
1	1st	Neg	Neg	Pos	A negative result from the first blood sample may be due to recent infection and IgM titre has not risen to the detectable level.
	2nd	Pos	Pos		
2	1st	Pos	Neg	Pos	A detectable IgM titre, without IgG, in the first sample indicate recent infection (likely 7–10 days ago). IgM antibodies in some horses could persist for at least 5 weeks and potentially longer [25]. Therefore, detection of IgM + IgG combination may indicate previous infection, rather than recently infected.
	2nd	Neg/Pos	Pos		
3	1st	Pos	Pos	Pos	
	2nd	Neg/Pos	Pos		
4	1st	Neg	Pos	Pos	Indicate previously infected. Not recent infection.
	2nd	Neg	Pos		
5	1st	Neg	Neg	Pos	It is unlikely that the patient or horse seroconverted to IgG, without IgM, upon recent infection. Results from the first sample is possibly due to false negative result in either IgG or IgM. Therefore, inconclusive as to recent or previous infection.
	2nd	Neg	Pos		
6	1st	Neg	Neg	Neg	N/A
	2nd	Neg	Neg		

Ig = Immunoglobulin; Pos = Positive; Neg = Negative; N/A = Not applicable; ^1^ Sample 1 and 2 should be collected at least 2–4 weeks apart.
